# Validation of online delivery of the Australian Pelvic Floor Questionnaire in an Irish obstetric population

**DOI:** 10.1007/s00192-023-05529-x

**Published:** 2023-04-01

**Authors:** Bobby D. O’Leary, Declan P. Keane

**Affiliations:** 1 Department of Urogynaecology, National Maternity Hospital, Holles Street, Dublin 2, Ireland; 2 UCD Perinatal Research Centre, National Maternity Hospital, Holles Street, Dublin 2, Ireland; 3 Royal College of Surgeons in Ireland, National Maternity Hospital, Holles Street, Dublin 2, Ireland

**Keywords:** Pelvic floor disorders, Labor, Questionnaires, Epidemiology, Assessment

## Abstract

**Introduction and hypothesis:**

Childbirth remains an important risk factor for the development of pelvic floor disorders, regardless of the mode of delivery. To accurately assess these symptoms, accurate, woman-centric assessments are needed. Online versions of these assessments may be especially useful in the COVID-19 era. Women may potentially answer questions differently in an online format, and this study aimed to validate an online version of the paper-based self-administered Australian Pelvic Floor Questionnaire (APFQ).

**Methods:**

The questionnaire was completed antenatally and at 3 months postpartum by 647 and 481 women respectively. Test– validity was assessed in subgroups of 61 and 57 women in each period, using intraclass correlation coefficients and Cohen’s kappa. Sensitivity to change was assessed by comparing responses during pregnancy to those at 3 months postpartum. Internal consistency was assessed using Cronbach’s alpha. Construct validity was assessed by comparing women with and without subjective bothersomeness.

**Results:**

Intraclass correlation coefficients were above 0.9 for all domains and the overall questionnaire. Cohen’s kappa for individual questions ranged from 0.71–1.00 across the antenatal and postnatal questionnaires. Cronbach’s alpha was acceptable for all domains except the prolapse domain. The APFQ was sensitive to changes occurring between antenatal recruitment and 3 months postpartum. Effect sizes ranged from 0.83–7.99.

**Conclusions:**

This online version of the APFQ is valid for assessing pelvic floor disorders in an Irish obstetric population. The APFQ is reproducible and responsive to change occurring with childbirth, and can be used to research longitudinal changes in pelvic floor disorders. As an online tool, this questionnaire may be useful in increasing response rates to clinical research.

## Introduction

Pelvic floor disorders remain common diseases in women and are expected to only increase with time [1–3]. Accurate, woman-centric assessments of pelvic floor disorders are thus required for both healthcare professionals and administrators to plan future healthcare provision. Several of these questionnaires exist [4–6], though they range in practicality for use in the routine antenatal clinic of a busy maternity hospital.

The Australian Pelvic Floor Questionnaire (APFQ) has already been validated in both interviewer and self-administered versions for use in urogynecology [7], and recently in the obstetric population [8]. The APFQ allows for the assessment of all pelvic floor symptoms, their impact on a woman’s quality of life, and the level of bother for the women concerned.

Several validated, paper-based English-language pelvic floor questionnaires are in existence, though none have been validated for use online. Research participants are most hesitant to return to healthcare settings in the setting of the COVID-19 pandemic [9]; thus, online delivery of questionnaires such as the APFQ may help increase response rates in research into female pelvic floor disorders. Women may potentially answer questions differently in an online format, and this study aimed to validate an online version of the paper-based self-administered APFQ.

## Methods and materials

This was a subgroup analysis of a prospective cohort study [10]. In short, women were recruited from consecutive antenatal clinics between May and October 2020, if they had a live, singleton fetus, and were 18 years or older. Those women with previous bladder or bowel surgery were excluded. There were no exclusion criteria based on parity. Women completed the paper-based APFQ whilst in the clinic, and were sent a reminder email with a link to the online version of the APFQ at 3 months postpartum.

The online-administered version of the validated APFQ contains the same questions but was formatted in an online-friendly presentation with automatic scaling for mobile devices through *Google Forms* (Google, Mountain View, CA, United States). Questions concerning bladder (15), bowel (12), sexual function (10), and pelvic organ prolapse symptoms (five) were grouped according to the physiological functions of the pelvic floor: bladder function, bowel function, prolapse symptoms, and sexual function domains. As with the offline, paper-based questionnaire, quality-of-life measures and bother scores are integrated into the four domains.

As the APFQ has been previously validated for face validity and convergent validity, and as the questions in the online version were identical to that of the paper version, these were not assessed.

### Test–retest reproducibility

Women initially completed the already-validated paper version of the APFQ after recruitment in the antenatal clinic. The online-administered pelvic floor questionnaire was completed by 481 women 3 months after delivery. A sample size calculation was performed to assess the test–retest reliability of pelvic floor disorder scores based on an anticipated intraclass correlation coefficient (ρ) of 0.9 [7]. To achieve a power of 80% and α = 0.05, 61 women were required [11]. The online questionnaire was completed a second time by a randomly selected subset of 61 women, 7 to 14 days after their administration of the paper version of the pelvic floor questionnaire. These same women were asked to complete the postnatal questionnaire a second time at the same 7–14-day time interval, such that these women would complete the online postnatal questionnaire twice.

Individual questions (categorical responses) were assessed using raw agreement (% answers identical) and Cohen’s kappa (raw agreement adjusted for random chance). A kappa value above 0.80 demonstrates excellent agreement between methods [12]. Bland–Altman plots with associated 95% limits of agreement and histograms [13] were generated for each domain and the overall pelvic floor scores in both sets of questionnaires, to assess for any fixed bias or outliers in the agreement between the paper and online questionnaires.

### Internal consistency

Given the breadth of symptoms assessed in each domain, we did not expect high consistency for all items. Regardless, internal consistency for each domain—bladder, bowel, prolapse, and sexual function—was assessed using Cronbach’s alpha. An alpha value above 0.70 is generally considered satisfactory [14].

### Sensitivity to change

Longitudinal follow-up of the 481 women who participated in both the antenatal and postnatal questionnaires was used to assess sensitivity to change. Effect size (ES, mean change of score/standard deviation of baseline score) and standardized response mean (SRM, mean change of score/standard deviation of the change of score) [15] were used to demonstrate the degree of responsiveness.

### Construct validity

Construct validity was assessed by testing if the questionnaire could discriminate between women with and without subjective bother scores, both in the antenatal and postnatal period. Domain scores were compared based on these groups and compared against the previously published minimal important difference (MID) of the APFQ [16].

### Statistical analyses

All statistical analyses were performed using R4.0.1 (R Foundation for Statistical Computing, Vienna, Austria). Demographics and scores were tested for normality, visually using histograms and via the Shapiro–Wilk test. Non-normal variables were summarized as medians with corresponding ranges, while normal variables were summarized as means (± standard deviations). Categorical variables are presented as *n*/*N* (%).

### Ethical approval

Ethical approval for this project was granted by the National Maternity Hospital Research and Ethics Committee (ref: EC 11.2019).

## Results

In total, 647 women answered the paper-based antenatal questionnaire and 481 completed the online postnatal questionnaire. Of the 647 women who answered the antenatal questionnaire, 61 (9.4%) completed the online re-test questionnaire. In the postnatal period, 57 (11.9%) women completed an online re-test questionnaire. Twenty women delivered elsewhere and so their obstetric and demographic information was not available, though none of these women responded to the postnatal questionnaire. Of the remaining 627 women, 45% were nulliparous, 42% were multiparous, and 13% had previous cesarean deliveries only. Two-thirds of women delivered vaginally, with 56% having a spontaneous delivery. Half of the women (49%) were overweight or obese. Demographic and obstetric information of those who completed the original questionnaire and the antenatal re-test questionnaire are summarized in Table 1. Women who completed the re-test questionnaire were more likely to be obese [21.3% (13/61) vs 11.5% (65/566), *p* = 0.045], but otherwise there were no demographic differences between the two groups.

**Table 1:** Comparison of women who completed the re-test questionnaire study and those who completed the paper version only

	Re-test group (*n* = 61)	Other participants (*n* = 566)	*P*
Maternal age	33.8 ± 5	33.2 ± 4.7	.337^a^
Gestation at delivery	40.1 (37.9–42.3)	40 (34.3–42.3)	.572^b^
Parity			
Primiparous	50.8% (31/61)	44.5% (252/566)	.584^c^
Previous vaginal deliveries	36.1% (22/61)	42.8% (242/566)	
Previous CS only	13.1% (8/61)	12.7% (72/566)	
Body mass index	26.6 ± 5.5	26.1 ± 4.9	.466^a^
Underweight	6.6% (4/61)	4.8% (27/566)	.145^c^
Normal	42.6% (26/61)	46.3% (262/566)	
Overweight	21.3% (13/61)	31.1% (176/566)	
Class I obesity	21.3% (13/61)	11.5% (65/566)	
Class II/III obesity	8.2% (5/61)	6.4% (36/566)	
Length of second stage	23 (3–154)	22 (0–199)	.969^b^
Prolonged second stage (> 120 min)	6.6% (4/61)	5.7% (32/566)	1^c^
Birthweight	3459 ± 526	3581 ± 514	.090^a^
Macrosomia	11.5% (7/61)	18.4% (104/566)	.244^c^
Mode of delivery			
Spontaneous	47.5% (29/61)	56.5% (320/566)	.315^d^
Vacuum	8.2% (5/61)	8.1% (46/566)	
Forceps*	0.0% (0/61)	2.8% (16/566)	
Cesarean in labour	19.7% (12/61)	13.3% (75/566)	
Pre-labour cesarean	24.6% (15/61)	19.3% (109/566)	
Perineal trauma			
Intact	50.8% (31/61)	46.5% (263/566)	.544^d^
1st degree	14.8% (9/61)	9.9% (56/566)	
2nd degree	19.7% (12/61)	26.7% (151/566)	
Episiotomy	13.1% (8/61)	15.4% (87/566)	
OASI**	1.6% (1/61)	1.6% (9/566)	

Continuous data presented as mean ± standard deviation or median (range). Categorical data presented as % (*n* / total)

^*^ Includes sequential instrumental deliveries ** Obstetric anal sphincter injury

^a^Student's *t*-test

^b^Mann–Whitney U-test

^c^Chi-square

^d^Fisher’s exact test

### Test–retest reproducibility

#### Antenatal questionnaire

**Fig. 1:**
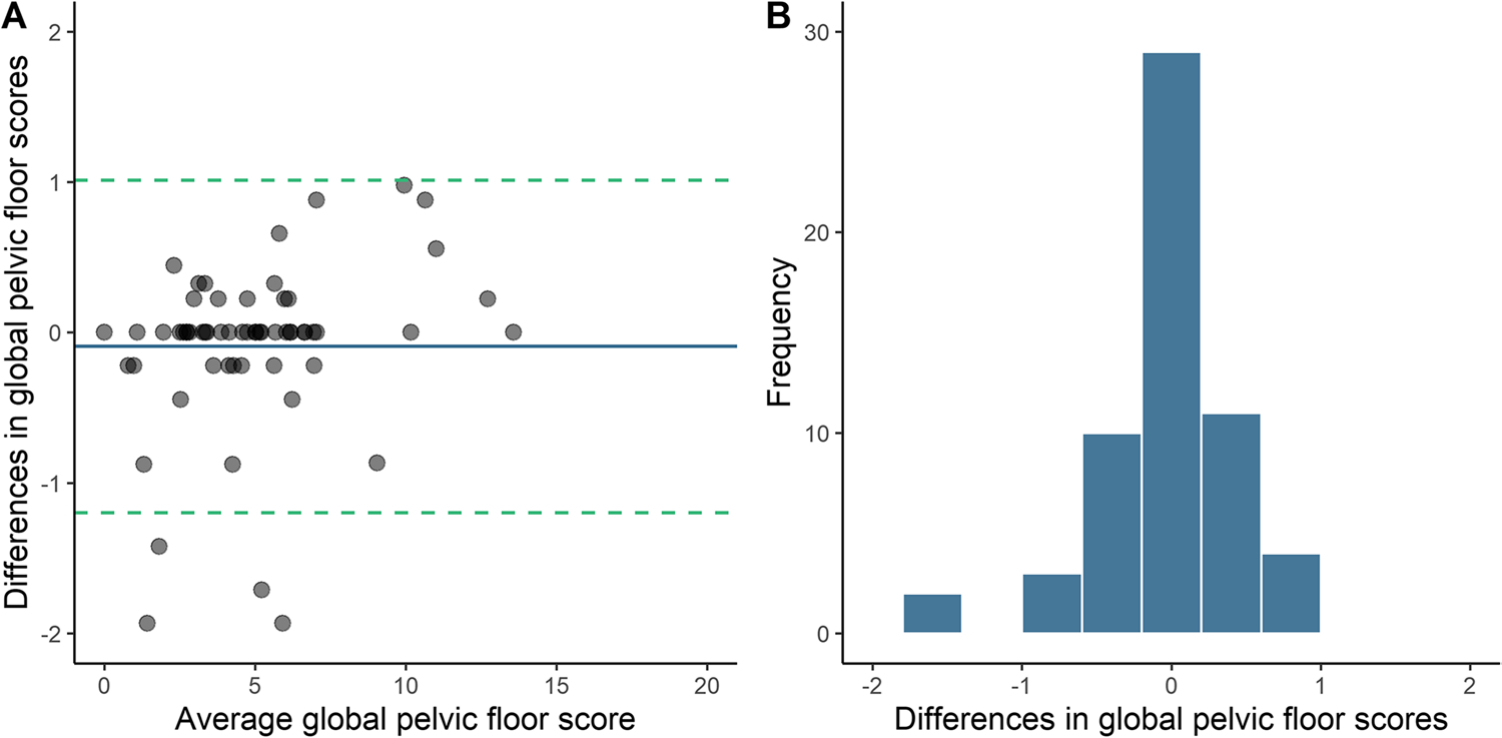
Bland–Altman plot (**A**) and histogram (**B**) for antenatal global pelvic floor scores

Of the 647 women who answered the antenatal paper questionnaire, 61 (9.4%) completed the online questionnaire approximately 1 week later. The replies to the second questionnaire were identical to the first in 84–98% of questions, and for those women who did change their answers; all were within one category. Intraclass correlation coefficients were above 0.9 for all domains and the overall questionnaire. For individual questions, the lowest kappa values were seen for the presence of a lump or bulge in the vagina (κ = 0.74) and laxative use (κ = 0.75). All other kappa values were above 0.80. Bland–Altman plots and histograms were generated to compare the test–retest scores for total pelvic floor scores (see Fig. 1). The mean difference (standard error of the mean) in the total pelvic floor disorder score between the two questionnaires was −0.09 (0.07). More than 93% (57/63) of values were within the 95% limits of agreement for total pelvic floor scores, and these differences appeared uniform across all total pelvic floor scores. Similar values were seen for each domain (data not shown). Mean differences (standard error of the mean) for the bladder, bowel, prolapse, and sexual function domains between the scores were −0.04 (0.02), −0.01 (0.03), −0.04 (0.02), and −0.01 (0.03) respectively, which we considered clinically non-significant.

#### Postnatal questionnaire

**Fig. 2:**
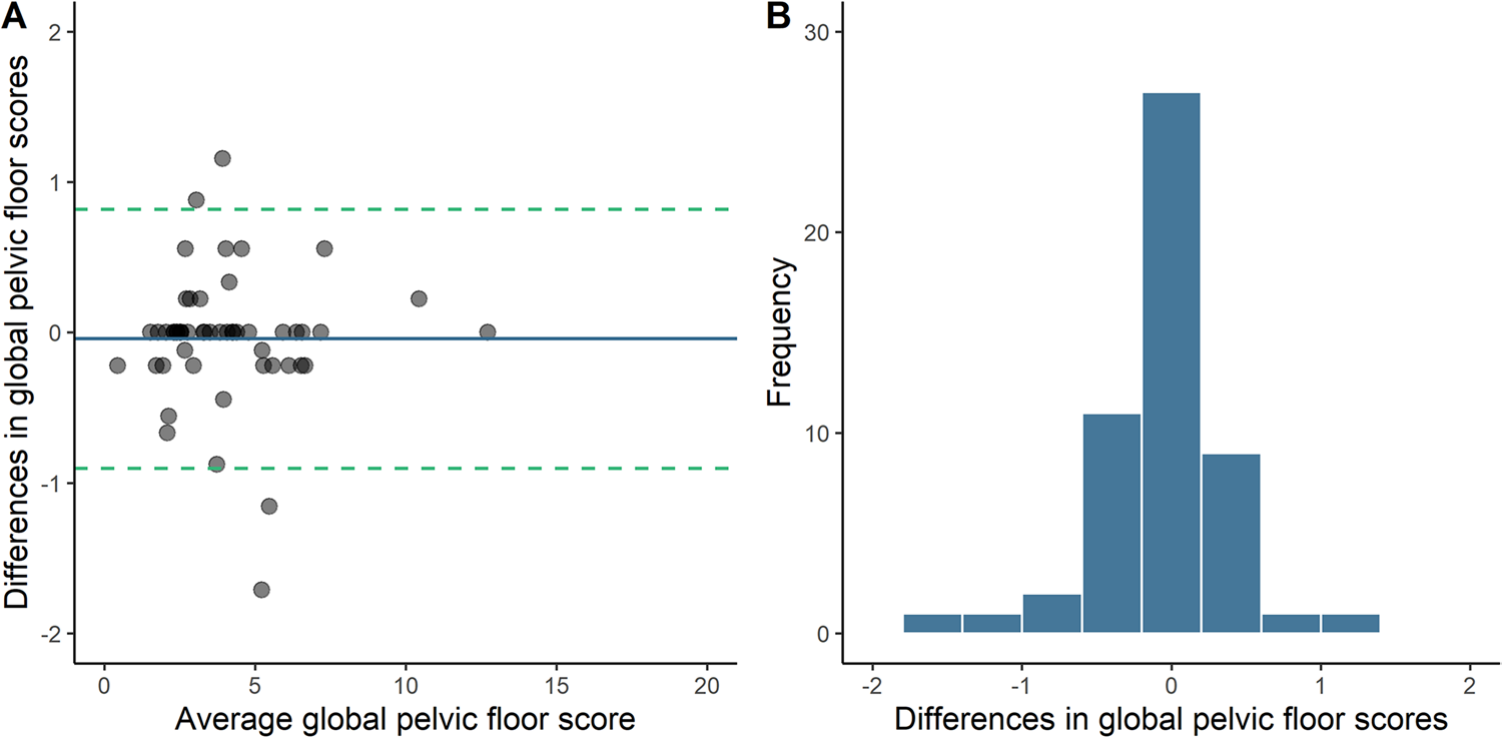
Bland–Altman plot (**A**) and histogram (**B**) for postnatal global pelvic floor scores

Of the 61 women invited to repeat the postnatal questionnaire, fifty-three responded (86.9%). Between 85 and 100% of women replied identically to the questions on the first and the repeat online postnatal questionnaires. Of those who did change their answer, all were within one category. Intraclass correlation coefficients were above 0.9 for all domains and the overall questionnaire. All kappa values in the test–retest analyses were above 0.70, with the lowest being 0.71 for the requirement to wear pads due to urinary incontinence. Bland–Altman plots were generated for total pelvic floor disorder scores, see Fig. 2. The mean difference (standard error of the mean) in the total pelvic floor disorder score between the two questionnaires was −0.04 (0.06). Over 92% (49/53) of differences in scores were between the 95% limits of agreement for the total pelvic floor score, and the differences in scores appeared uniform across the full range of scores for each of the domains and the total score. Similarly, high levels of agreement were seen for each of the individual domains (data not shown). Mean differences (standard error of the mean) for the bladder, bowel, prolapse, and sexual function domains between the scores were −0.06 (0.02), 0.01 (0.02), −0.02 (0.03), and 0.03 (0.03) respectively, which, like the antenatal scores, was considered not to be clinically significant.

#### Internal consistency

Internal consistency was only assessed for the postnatal questionnaire as the self-administered version has been previously validated. Cronbach’s alpha for the four domains in the postnatal questionnaire was: bladder domain 0.76, bowel domain 0.65, prolapse domain 0.59, and sexual function domain 0.63.

#### Sensitivity to change

There were significant differences between antenatal and postnatal scores in all domains except prolapse. This was reflected in the effect size and standardized response mean for each domain and the overall pelvic floor score (see Table 2). Overall, the APFQ showed excellent responsiveness to change at 3 months postpartum.

**Table 2:** Sensitivity to change across individual domains and total pelvic floor scores

	Scores			Responsiveness		
Domain	Antenatal score	Postnatal score	Difference	Effect size	Standardised response mean	Wilcoxon signed rank test
Bladder	0.4 (0–2.4)	1.3 (0–6.7)	0.8 (0–4.3)	2.51	1.32	< .001
Bowel	0.5 (0–2.4)	1.6 (0–5.2)	1.1 (0–3.9)	3.21	1.57	< .001
Prolapse	0 (0–2.2)	0 (0–5)	0 (0–3.5)	0.83	0.50	.732
Sexual function	0.1 (0–1.5)	2.8 (0–8.9)	2.2 (0–8.3)	7.99	1.49	< .001
Total	1.3 (0–5.7)	5.7 (0–19.8)	4.3 (0–14.4)	4.83	1.63	< .001

Data presented as median (range)

#### Construct validity

The postnatal questionnaire significantly distinguished the symptom scores between women with and without subjective suffering bothersome symptoms (*p* < 0.001 for all domains), (see Table 3). Women who reported “slightly”, “moderately” and “greatly” bother in four domains in the antenatal period and at 3 months postpartum had significantly higher symptom scores compared to women who reported “not at all”. The mean difference between each group exceeded the MID for all domains.

**Table 3:** Score differences between women with and without subjective bother scores antenatally and at 3 months postnatal

Domain	Time	Bother*	*N*	Score	*P*^†^
Bladder	Antenatal	NO	307	0.3 (0.0–1.8)	< .001
		YES	174	0.8 (0.2–2.4)	
	Postnatal	NO	280	0.9 (0.0–4.9)	< .001
		YES	201	2.0 (0.0–6.7)	
Bowel	Antenatal	NO	311	0.4 (0.0–1.5)	< .001
		YES	170	0.7 (0.1–2.4)	
	Postnatal	NO	293	1.0 (0.0–3.2)	< .001
		YES	188	2.3 (0.0–5.2)	
Prolapse	Antenatal	NO	447	0.0 (0.0–1.7)	< .001
		YES	34	1.0 (0.2–2.2)	
	Postnatal	NO	441	0.0 (0.0–5.0)	< .001
		YES	40	1.7 (0.0–5.0)	
Sexual function	Antenatal	NO	369	0.1 (0.0–1.2)	< .001
		YES	112	0.6 (0.3–1.5)	
	Postnatal	NO	308	2.2 (0.0–8.9)	< .001
		YES	173	2.8 (0.0–8.3)	

Scores presented as median (range)

^*^ Significant bother defined as any woman reporting a bother score of "slightly", "moderately" or "greatly"

^†^ Wilcoxon rank sum test

## Discussion

This online-administered version of the APFQ was shown to be reliable, reproducible, and sensitive to change in an Irish obstetric population. The development of this questionnaire in an online format may aid in further research studies, where women do not wish to return to complete the questionnaire or are unlikely to return a paper-based questionnaire in the post. Analysis of antenatal and postnatal responses shows that this questionnaire is sensitive to change following childbirth.

A recent study of 316 Chinese women validated the APFQ for use in the 3rd trimester and 5–6 months postpartum [8]. Similar to our results, the internal consistency scores and intraclass correlation coefficient for the prolapse domain were poor. Despite this, women who reported subjective bother associated with prolapse had significantly higher scores than those reporting no bother. In contrast to our work, the prolapse domain in the Chinese study remained sensitive to change. This difference between our two studies may relate to a difference in language or cultural acceptance of prolapse symptoms. All other domains were internally consistent and sensitive to change, as seen in the present analysis. Effect sizes were larger in our study, though this may be due to earlier responses in the postpartum period (3 months vs 6 months) as well as because some of our women were recruited earlier in pregnancy compared to those in China.

The self-administered APFQ was initially validated in an Australian urogynecology setting, which may have given different results compared to our younger obstetric population. Additionally, this validation was carried out with a distinct, measurable intervention—pelvic floor surgery. Childbirth is significantly different in that multiple factors are affecting postpartum pelvic floor function, and so cannot be distilled down to *intervention* and *control*. Larger effect sizes and standardized response means were seen in our cohort, though this may be due to the lower baseline scores in our group and because childbirth may have a larger impact on pelvic floor disorders than a urogynecological procedure does.

There is potential awkwardness in disclosing pelvic floor disorder, especially symptoms such as urinary and fecal incontinence. Self-administered questionnaires give women space for more ‘honest’ responses, and a further degree of separation via an online portal may allow for more candid reporting [17]. Additionally, the COVID-19 pandemic has heightened—and perhaps rightfully so—many service users’ fears of attending the extra appointments involved in research. Thus, the online implementation of a questionnaire may improve participant confidence in any research study, and hopefully improve response rates.

### Strengths and limitations

We feel our results are robust for several reasons. While women did not undergo a specified intervention such as a pelvic floor repair, their questionnaire responses were captured at two physiologically distinct time points—during pregnancy and 3-months after childbirth—and the questionnaire exhibited significant sensitivity to change. Similarly, we had a sizable number of women complete the test–retest questionnaires at both time points, and the overall sample size of our study was twice that of that performed previously [8]. Internal consistency scores were comparable to those reported in China [8] and Australia [7], suggesting that pregnant women in Ireland gave similar responses to the questionnaire. Finally, this analysis represents a validated online questionnaire for measuring PFD in pregnant women, and highlights the potential of online delivery of such questionnaires for use in research in the COVID-19 era.

There are some limitations worthy of discussion. The internal consistency of the prolapse domain was poor; and while Cohen’s Kappa values and ICC for this domain were satisfactory, it was not sensitive to change and thus may not be suitable for use in assessing prolapse symptoms in the pregnant woman. While vaginal childbirth [18, 19] has been strongly associated with prolapse, it is entirely possible that these symptoms have simply not begun at this time, and may appear later in these women’s lives. Although quality-of-life measures are included in each domain, if a comprehensive assessment of the impact of pelvic floor disorders on a woman’s quality of life is required, the addition of a dedicated questionnaire such as the ICI-Vaginal Symptoms [6] assessment or the Pelvic Floor Impact Questionnaire [20] may be more appropriate. Our study was limited to women attending the routine antenatal clinics of a single maternity hospital in the East of Ireland, and this may have introduced some degree of selection bias. As with all questionnaire studies, women may have had personal reasons to partake—or not—and so there will be a degree of response bias in our results. This will be present in any research in this area and is not possible to account for. Recording the reason why women did not take part was limited by the ethical approval of this study, though in practice this number was small. Further research should include reason for refusal, if possible. Third, women who took part in the test–retest questionnaires—while randomly selected—may also have had reasons for wishing to take part that could have influenced our results. Women who took part were essentially identical to those who did not on a demographic level; however, we cannot adjust for any undetected differences. Similar to other research in this area, recruitment of women in antenatal clinic settings may underrepresent some ethnic minorities or women in whom English is not their first language [21]. Fourth, construct validity was not assessed using a further questionnaire, though the symptoms-based approach is the same as that used by Hou and Hou [8] while validating the APFQ in China. While we had an appropriate number of women for the initial antenatal test–retest cycle, we had a small drop-off for the postnatal cycle, and this may have affected our ability to assess test–retest validity in the postnatal questionnaire. Finally, the language of the sexual function domain questions suggests a heterosexual relationship—a limitation raised by several participants—and thus may not accurately capture sexual dysfunction for all women. Patient and public involvement may help to direct any further modifications of the APFQ in the future in this regard.

### Conclusion

The APFQ can be utilized in an online format in an Irish obstetric population and is sensitive to the changes that occur in pelvic floor symptoms following childbirth. Online delivery of research interventions such as questionnaires may lead to a higher number of responses, and may allow women to disclose potentially more embarrassing symptoms compared to a face-to-face interview. Further research into online formats of other pelvic floor questionnaires is warranted.

## References

[1] Mellgren A, Jensen LL, Zetterström JP et al. Long-term cost of fecal incontinence secondary to obstetric injuries. Dis Colon Rectum. 1999;42:857–65. Discussion 865–7 10.1007/BF02237089 10411431

[2] Dunivan GC, Anger JT, Alas A (2014). Pelvic organ prolapse: a disease of silence and shame. Female Pelvic Med Reconstr Surg.

[3] DeLancey JOL (2005). The hidden epidemic of pelvic floor dysfunction: achievable goals for improved prevention and treatment. Am J Obstet Gynecol.

[4] Avery KNL, Bosch JLHR, Gotoh M (2007). Questionnaires to assess urinary and anal incontinence: review and recommendations. J Urol.

[5] Zuchelo LTS, Bezerra IMP, Da Silva ATM (2018). Questionnaires to evaluate pelvic floor dysfunction in the postpartum period: a systematic review. Int J Womens Health.

[6] Abrams P, Avery K, Gardener N (2006). The International Consultation on Incontinence Modular Questionnaire: www.iciq.net. J Urol.

[7] Baessler K, O’Neill SM, Maher CF, Battistutta D (2010). A validated self-administered female pelvic floor questionnaire. Int Urogynecol J.

[8] Hou Y, Hou D (2020). Validation of the Australian Pelvic Floor Questionnaire in Chinese pregnant and postpartum women. Eur J Obstet Gynecol Reprod Biol.

[9] Padala PR, Jendro AM, Gauss CH (2020). Participant and caregiver perspectives on clinical research during Covid-19 pandemic. J Am Geriatr Soc.

[10] O’Leary BD, Keane DP (2022). The effect of the length of the second stage of labor on pelvic floor dysfunction. Am J Obstet Gynecol MFM.

[11] Zou GY (2012). Sample size formulas for estimating intraclass correlation coefficients with precision and assurance. Stat Med.

[12] Koch GG, Landis JR, Freeman JL (1977). A general methodology for the analysis of experiments with repeated measurement of categorical data. Biometrics.

[13] Bland JM, Altman DG (2003). Applying the right statistics: analyses of measurement studies. Ultrasound Obstet Gynecol.

[14] Bland JM, Altman DG (1997). Cronbach’s alpha. BMJ.

[15] Verdam MGE, Oort FJ, Sprangers MAG (2017). Structural equation modeling–based effect-size indices were used to evaluate and interpret the impact of response shift effects. J Clin Epidemiol.

[16] Baessler K, Mowat A, Maher CF (2019). The minimal important difference of the Australian Pelvic Floor Questionnaire. Int Urogynecol J.

[17] Davis RN (1999). Web-based administration of a personality questionnaire: comparison with traditional methods. Behav Res Methods Instrum Comput.

[18] DeLancey JOL, Morgan DM, Fenner DE (2007). Comparison of levator ani muscle defects and function in women with and without pelvic organ prolapse. Obstet Gynecol.

[19] Dietz HP, Simpson JM (2008). Levator trauma is associated with pelvic organ prolapse. BJOG.

[20] Barber MD, Kuchibhatla MN, Pieper CF, Bump RC (2001). Psychometric evaluation of 2 comprehensive condition-specific quality of life instruments for women with pelvic floor disorders. Am J Obstet Gynecol.

[21] van Delft K, Schwertner-Tiepelmann N, Thakar R, Sultan AH (2013). Recruitment of pregnant women in research. J Obstet Gynaecol.

